# The Fusion Loops of the Initial Prefusion Conformation of Herpes Simplex Virus 1 Fusion Protein Point Toward the Membrane

**DOI:** 10.1128/mBio.01268-17

**Published:** 2017-08-22

**Authors:** Juan Fontana, Doina Atanasiu, Wan Ting Saw, John R. Gallagher, Reagan G. Cox, J. Charles Whitbeck, Lauren M. Brown, Roselyn J. Eisenberg, Gary H. Cohen

**Affiliations:** a Faculty of Biology and Astbury Centre for Structural Molecular Biology, University of Leeds, Leeds, United Kingdom; b Department of Microbiology, School of Dental Medicine, University of Pennsylvania, Philadelphia, Pennsylvania, USA; c Department of Pathobiology, School of Veterinary Medicine, University of Pennsylvania, Philadelphia, Pennsylvania, USA; Columbia University Medical College

**Keywords:** cryo-electron microscopy, cryo-electron tomography, HSV, subtomogram averaging, gB, herpesviruses, microvesicles, neutralizing antibodies, prefusion, viral fusion

## Abstract

All enveloped viruses, including herpesviruses, must fuse their envelope with the host membrane to deliver their genomes into target cells, making this essential step subject to interference by antibodies and drugs. Viral fusion is mediated by a viral surface protein that transits from an initial prefusion conformation to a final postfusion conformation. Strikingly, the prefusion conformation of the herpesvirus fusion protein, gB, is poorly understood. Herpes simplex virus (HSV), a model system for herpesviruses, causes diseases ranging from mild skin lesions to serious encephalitis and neonatal infections. Using cryo-electron tomography and subtomogram averaging, we have characterized the structure of the prefusion conformation and fusion intermediates of HSV-1 gB. To this end, we have set up a system that generates microvesicles displaying full-length gB on their envelope. We confirmed proper folding of gB by nondenaturing electrophoresis-Western blotting with a panel of monoclonal antibodies (MAbs) covering all gB domains. To elucidate the arrangement of gB domains, we labeled them by using (i) mutagenesis to insert fluorescent proteins at specific positions, (ii) coexpression of gB with Fabs for a neutralizing MAb with known binding sites, and (iii) incubation of gB with an antibody directed against the fusion loops. Our results show that gB starts in a compact prefusion conformation with the fusion loops pointing toward the viral membrane and suggest, for the first time, a model for gB’s conformational rearrangements during fusion. These experiments further illustrate how neutralizing antibodies can interfere with the essential gB structural transitions that mediate viral entry and therefore infectivity.

## IMPORTANCE

The herpesvirus family includes herpes simplex virus (HSV) and other human viruses that cause lifelong infections and a variety of diseases, like skin lesions, encephalitis, and cancers. As enveloped viruses, herpesviruses must fuse their envelope with the host membrane to start an infection. This process is mediated by a viral surface protein that transitions from an initial conformation (prefusion) to a final, more stable, conformation (postfusion). However, the prefusion conformation of the herpesvirus fusion protein (gB) is poorly understood. To elucidate the structure of the prefusion conformation of HSV type 1 gB, we have employed cryo-electron microscopy to study gB molecules expressed on the surface of vesicles. Using different approaches to label gB’s domains allowed us to model the structures of the prefusion and intermediate conformations of gB. Overall, our findings enhance our understanding of HSV fusion and lay the groundwork for the development of new ways to prevent and block HSV infection.

## INTRODUCTION

Herpes simplex virus (HSV) is a model system for the herpesvirus family, which includes human viruses that cause lifelong infections and a variety of diseases, including skin lesions, encephalitis, and cancers. HSV, which is categorized into two types (HSV-1 and HSV-2), also causes a highly contagious infection common and endemic throughout the world. It is estimated that over 3.5 billion people worldwide are infected with HSV-1, while over 400 million people are infected with HSV-2, an infection that has been shown to increase the risk of HIV acquisition (1). Antivirals that reduce the severity and frequency of HSV symptoms exist. However, these drugs cannot cure infection and there is no HSV vaccine available.

A key step of viral infection is entry into the host cell, a process that for enveloped viruses like HSV involves fusion of viral and cellular membranes, allowing the viral genome to access the interior of the cell. Enveloped virus fusion is mediated by viral transmembrane proteins, and mounting evidence suggests that these proteins have converged on a similar overall strategy among different viruses and classes of fusion proteins (2). Herpesvirus entry and membrane fusion require three virion glycoproteins that function as the “core fusion machinery,” gB, the actual fusion protein, and the gH/gL heterodimer (3). Additionally, HSV fusion requires the gD glycoprotein (4). Atomic models for many of the HSV glycoproteins exist, including for gD in its unliganded form (5) and in complex with its receptors (6–9); for a partially activated gH/gL complex (10); and for the postfusion form of gB (11, 12). Structures of Epstein-Barr virus gH/gL alone and in complex with gp42 (13, 14) and gB (15), human cytomegalovirus gB (16, 17), pseudorabies virus gH/gL (18), and varicella-zoster virus gH/gL (19) are also available.

Current HSV fusion models propose that receptor-activated gD converts the regulatory protein gH/gL to an active state, which in turn promotes the fusogenic ability of gB, the fusion protein (20). A detailed description of this process is reviewed elsewhere (4, 21). Of note, Rogalin and Heldwein have recently generated vesicular stomatitis virus (VSV) particles pseudotyped with HSV-1 gD, gH/gL, and gB, and these particles were found to be able to infect cells expressing gD’s receptor, showing that gD, gH/gL, and gB are not only essential but also presumably sufficient for HSV cell entry (22).

**FIG 1:**
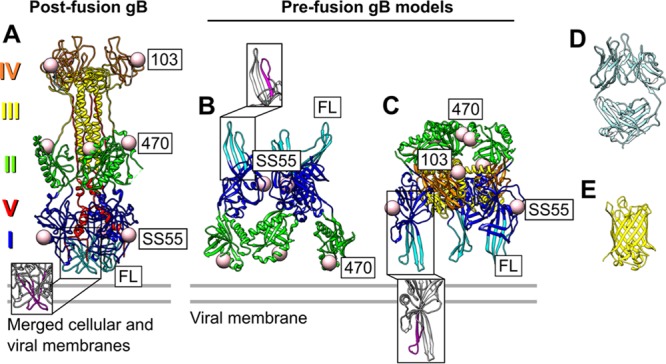
Pre- and postfusion models. (A to C) The atomic structure of postfusion gB (A) (12) is shown together with the gB prefusion models of Zeev-Ben-Mordehai et al. (B) (30) and Gallagher et al. (C) (34). The domains are color coded as follows. Domain I, containing the fusion loops (FL) in cyan, is blue; domain II is green; domain III is yellow; domain IV is orange; and domain V is red. The insets show the position of fusion loop 2 (residues 252 to 271, magenta). The locations of the FP insertions discussed here (position 103 is shown instead of 100 because 100 is not visible in the postfusion atomic structure), the binding region of SS55, and the fusion loops are labeled. Zeev-Ben-Mordehai et al. did not include domains III to V in their model, and Gallagher et al. did not include domain V. All of the models also lack the N terminus, which was disordered in the postfusion gB crystals, and amino acids 730 to 774, which were cleaved for gB postfusion purification purposes. Fab (D) and GFP (E) are shown for size comparison.

According to their structural features, viral fusion glycoproteins are sorted into three classes. HSV-1 gB is a 904-amino-acid class III fusion glycoprotein. The postfusion structure of gB was determined via X-ray crystallography of a truncated form ending at amino acid 730. Postfusion gB contains five structural domains composed of α-helices and β-sheets, of which three are discontinuous and inserted into other domains (Fig. 1A) (11, 12). Seen from the side, the trimeric postfusion gB appears as an elongated three-lobed structure. Domains I and V are at the “base” (close to the viral membrane) of the trimer (respectively, blue and red in Fig. 1). Domain I contains the fusion loops (cyan in Fig. 1) and is therefore referred to as the fusion domain. Domain II (green in Fig. 1) comprises the central lobe and mediates interactions with gH/gL, as evidenced by the fact that certain monoclonal antibodies (MAbs) that bind to this domain (23) block association with gH/gL (24). Domain III (yellow in Fig. 1) connects the central and top lobes and consists of α-helices that form a trimeric coiled coil. Domain IV (orange in Fig. 1) is at the top of postfusion gB (the “crown”) and might be involved in the interaction with a cellular receptor, as suggested by the fact that MAbs to this region block gB cell binding (25, 26). Finally, the postfusion gB structure lacks the N terminus (amino acids 31 to 102), which was flexible in the crystals, and amino acids 730 to 904 (containing the membrane-proximal region [MPR], transmembrane domain, and cytoplasmic tail), which were cleaved for purification and crystallization purposes. The structural domains of gB can also be grouped into four functional regions (FRs), according to the mapping of epitopes of a panel of neutralizing MAbs to distinct regions of the gB structure (27). According to this mapping, FR1 includes domains I and V, FR2 includes domain II, FR3 includes domain IV, and FR4 includes the unsolved N-terminal region.

While the structure of postfusion gB has significantly advanced our understanding of the fusion process of HSV, the structure of prefusion gB is essential in order to complete our knowledge of how gB undergoes this transition. Also, it is this form or an intermediate that must be the target of neutralizing antibodies against gB and that could provide rational ways to block its transition to the postfusion form that culminates in virus entry. However, solving the structure of prefusion gB has been hampered by the fact that all of the purified forms of gB are postfusion. Attempts to alter this outcome by modification of gB, e.g., point mutations, deletions, and truncations, have been unsuccessful (28). Additionally, HSV virions contain more than 12 different types of glycoproteins on their surface, making identification of the unknown prefusion conformation of gB very challenging (29).

To generate a model of prefusion gB, a protocol expressing full-length gB embedded in a lipid bilayer was developed, making it amenable to cryo-electron tomography (cryo-ET) and subtomogram averaging (30). The molecules expressed this way adopted two distinct conformations, one that corresponds to the elongated postfusion form of gB and another that corresponds to a compact form, putatively gB in a prefusion conformation. Constrained rigid-domain positioning of two of the five domains of truncated postfusion gB into the prefusion average allowed the generation of a pseudoatomic model of part of the putative prefusion form of gB (Fig. 1B). This model, from Zeev-Ben-Mordehai et al. (30), suggests that the fusion loops within domain I (cyan in Fig. 1) point away from the viral membrane. An alternative, and strikingly different, model of prefusion gB was proposed that is based on the similarities of gB and the VSV fusion protein (protein G), another class III fusion protein whose pre- and postfusion structures are known (31, 32). This model, from Gallagher et al. (33, 34), proposes that the fusion loops point down toward the virion envelope (Fig. 1C), as has been shown for prefusion VSV-G. Similar models with this orientation have been proposed for prefusion gB of the herpesviruses Epstein-Barr virus and human cytomegalovirus (15, 35). However, these *in silico* models lack experimental validation.

In this study, our goal was to attempt to reconcile these disparate models of prefusion gB. In particular, we augmented the approach of Zeev-Ben-Mordehai et al. by taking advantage of a system that produced microvesicles containing (i) full-length gB with embedded fluorescent proteins (FPs), (ii) gB coexpressed with neutralizing antibodies, and (iii) gB incubated with antibodies against the fusion loops. We hypothesized that visualizing these additional forms would allow us to discern which of the two current gB prefusion models is correct. We characterized the microvesicles expressing these gB forms by using cryo-ET for direct 3D imaging. We combined this technique with subtomogram averaging, which improves the signal-to-noise ratio of repeated structures within a tomogram. This approach made the FPs, Fabs, and antibodies visible, enabling us to use them as structural landmarks for localization of the different gB domains. Additionally, we showed that the different gB samples were antigenically intact, as determined by the fact that gB epitopes covering all gB domains were preserved when expressed in microvesicles. In summary, these experiments provide experimental support for the arrangement of the initial prefusion gB that resembles that of VSV-G and models proposing that the fusion loops point down toward the virion envelope, rather than away from the viral membrane. Thus, our data provide experimental evidence that the model proposed by Zeev-Ben-Mordehai et al. does not represent the starting prefusion conformation of gB, albeit it could certainly represent an intermediate. Our results also provide important insights into how the transition of gB from its prefusion state to its postfusion state takes place and therefore provide a starting point for a molecular understanding of the HSV fusion process and consequently for the development of new approaches to the prevention of HSV infection.

## RESULTS

### Expression of full-length gB.

To express full-length HSV gB in a lipid membrane, we used two different approaches, HIV pseudotyping and microvesicle generation. To carry out pseudotyping, we expressed HIV structural proteins in the absence of the viral genome, replacing the HIV glycoprotein with full-length gB (36). To generate microvesicles, it was sufficient to transfect 293T cells with the gene for full-length gB. This approach was first described for VSV-G (37) and has been used to produce microvesicles expressing gB and other membrane proteins on their surface (38).

**FIG 2:**
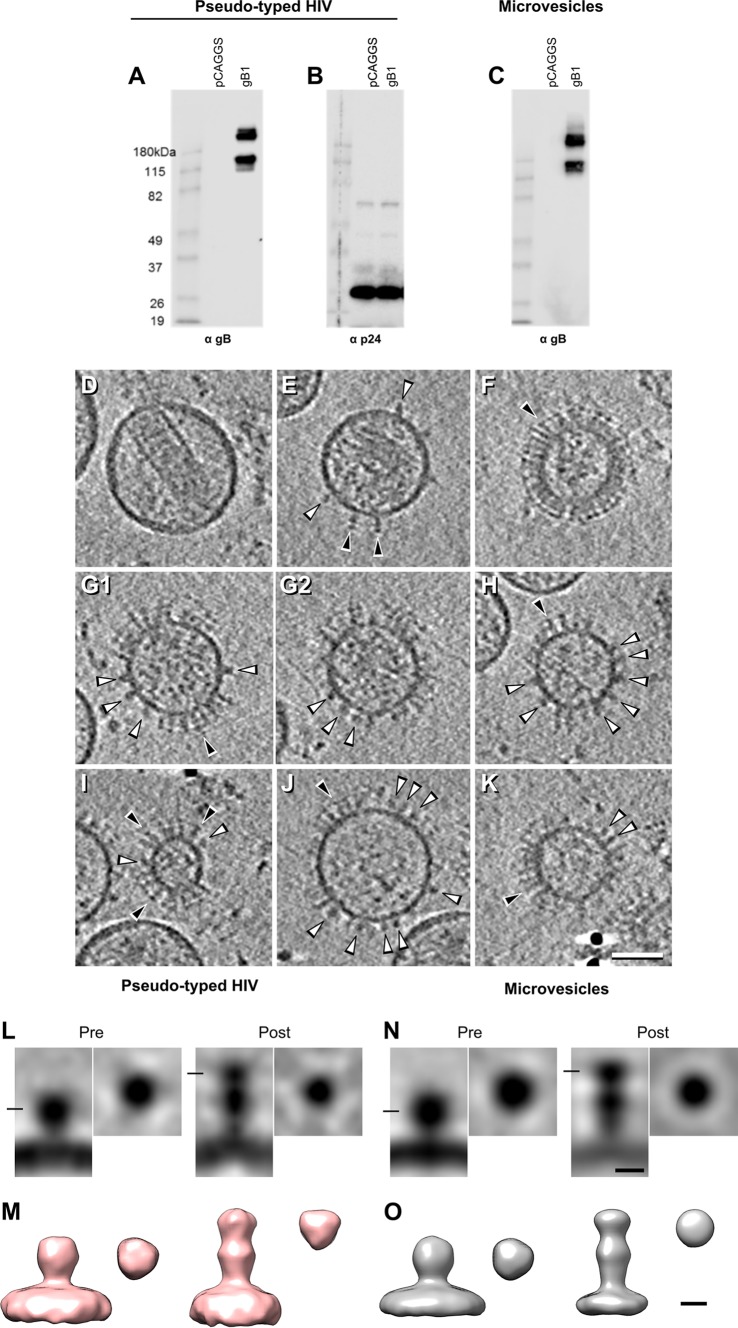
Characterization of pseudotyped HIV particles and microvesicles expressing full-length gB by Western blotting and cryo-ET and subtomogram imaging. (A to C) Pseudotyped HIV particles (A and B) or microvesicles (C) were isolated from transfected 293T cells. Equal volumes of vesicles from both preparations were loaded onto a 10% Tris-glycine gel under native conditions and probed with gB PAb R217. The presence of Gag protein in the HIV-like particles was confirmed with a MAb (B). (D to K) Central tomographic sections of HIV-like particles with few gB molecules (D and E) and of microvesicles containing many gB molecules (F to K). gB adopts two distinct conformations, postfusion gB (black arrowheads) and the compact prefusion form of gB (white arrowheads). (L to O) Subtomogram averaging of the two conformations of full-length gB in both types of samples. (L and M) Averages of HIV-like particles. (N and O) Averages of microvesicles. (L and N) Sections through the subtomogram averages of pre- and postfusion gB. For each of the four averages, sagittal sections are on the left and transverse sections are on the right (at the height of the line shown in the left panels). (M and O) Isosurface renderings of pre- and postfusion gB seen from the side (left) and top (right). Bars: D to K, 50 nm; L to O, 5 nm.

Both approaches produced vesicles expressing gB, as shown by Western blotting (Fig. 2A and C) and visually by electron microscopy (EM), as evidenced by the presence of postfusion gB molecules (Fig. 2D to K). The HIV pseudotyped particles also contained HIV-encoded proteins, as illustrated in a Western blot assay against HIV capsid protein p24 (Fig. 2B).

While few HIV particles displayed membrane-associated proteins (Fig. 2D and E), many were seen in the microvesicles (Fig. 2F to K). Importantly, membrane-associated proteins in both the HIV-like particles and microvesicles corresponded to two different structures, (i) an elongated form compatible with postfusion gB and (ii) a more compact form similar to that found by Zeev-Ben-Mordehai et al. (30) and compatible with the prefusion gB model proposed by Gallagher et al. (34). The average number of gB molecules per particle was 40 for microvesicles and 16 for HIV-like particles. In both types of particles, the majority of gB molecules, in a ratio of 5:1, adopted the postfusion conformation (11). To improve the signal-to-noise ratio of these structures, we performed subtomogram averaging for each gB conformation from each type of vesicle (Fig. 2L to O). The postfusion form of gB was approximately 18 nm tall and approximately 6 nm wide, and the crown and central lobe were easily recognized. The putative prefusion gB had a globular structure adjacent to the membrane that was approximately 8 nm tall and 7 nm wide. This form was identical in both pseudotyped HIV particles and microvesicles. Because of the higher yield and the simplicity of the system, we characterized and visualized the different gB forms expressed in microvesicles.

### Antigenicity of full-length gB tagged with FPs expressed in microvesicles.

To confirm that the compact structures observed on the surface of microvesicles were indeed gB, we took advantage of the fact that this protein tolerates the insertion of FPs at specific sites while maintaining its fusion capabilities (34). Since FPs should be visible in the subtomogram averages, we reasoned that they could be used to label gB and serve as landmarks to localize its specific domains. Additionally, we reasoned that this approach would allow us to distinguish between the two proposed gB prefusion models (Fig. 1B and C).

**FIG 3:**
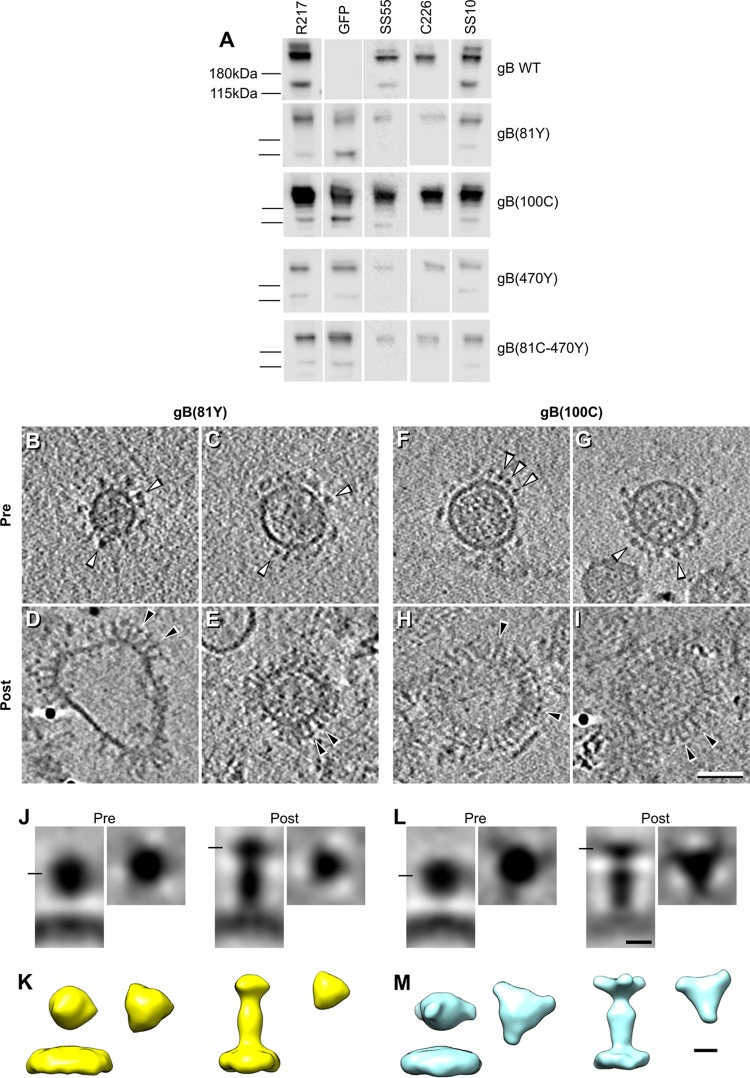
Antigenic characterization of full-length gB-FPs expressed in microvesicles. (A) Nondenaturing Western blot assay of WT gB, gB(81Y), gB(100C), gB(470Y), and gB(81C-470Y). Equal volumes of vesicles isolated from cells transfected with the gB constructs indicated were loaded onto a 10% Tris-glycine gel under native conditions and probed with gB (R217) and GFP PAbs. The presence of intact epitopes for major neutralizing MAbs (SS55, C226, SS10), which mapped different domains of gB, confirmed the integrity of gB molecules presented on microvesicles. (B to I) Central tomographic sections of microvesicles expressing full-length gB(81Y) (B to E) or gB(100C) (F to I). These microvesicles contained predominantly a single gB conformation, either compact gB (B, C, F, and G; white arrowheads) or postfusion gB (D, E, H, and I; black arrowheads). (J to M) Subtomogram averaging of pre- and postfusion gB(81Y) (J and K) and gB(100C) (L and M). The distribution of the panels is the same as that shown for WT gB in Fig. 2. Bars: B to I, 50 nm; J to M, 5 nm.

To assess the proper folding of the different gB samples, microvesicle preparations expressing full-length WT gB and the gB-FPs were characterized by “native” Western blotting of the proteins separated on nondenaturing gels (39) (Fig. 3A). All vesicle preparations were recognized by a gB polyclonal antibody (PAb), R217. As expected, only the gB-FP constructs reacted with a green FP (GFP) PAb. All gB constructs were also recognized by a set of potent neutralizing MAbs that were mapped to the different domains of gB, including SS55 (domain I), C226 (domain II), and SS10 (domain IV) (23). This suggests that all of the domains of full-length gB expressed on these vesicles were properly folded and all epitopes were correctly presented.

### Localization of the N-terminal domain expressing gB(81Y) and gB(100C).

We then used cryo-ET to study two gB-FP constructs, one containing a yellow FP (YFP) tag embedded at amino acid 81, gB(81Y), and another with a cyan FP embedded at amino acid 100, gB(100C) (34). Residues 81 and 100 are located near the N terminus of gB (in putative domain VI). As mentioned above, no structure is available for this region, because it is too flexible in postfusion gB crystals to be resolved (12). However, we hypothesized that, given their size, the FPs would locate the general position of domain VI and the beginning of domain IV (starting at residue 103). Indeed, the postfusion gB structure located residue 103 and the FP at the top of gB in the vesicles (Fig. 1A). In contrast, the prefusion gB model of Zeev-Ben-Mordehai et al. tentatively located it at the bottom of prefusion gB and close to the membrane (although they did not include it in their pseudoatomic model). In the model of Gallagher et al., domain IV is positioned at the upper middle of prefusion gB and faces outward (Fig. 1C).

Like the WT, the full-length gB(81Y) protein expressed on microvesicles adopted two conformations, a postfusion conformation and a compact conformation. However, in this sample, vesicles containing compact gBs were approximately four times as abundant as postfusion gB-containing vesicles (Fig. 3B to E). As expected, subtomogram averaging showed three small densities at the top of the molecule in postfusion gB (compare Fig. 1A with Fig. 3J and K, right panels). Unlike WT gB, the compact conformation of gB(81Y) (approximately 9 nm tall and 8 nm wide) was separated by approximately 4 nm from the membrane, with three small densities at the sides (Fig. 3J and K, left panels). We interpret the extra densities at the top of postfusion gB(81Y) and at the side of compact gB(81Y) to be the YFP tag, confirming our hypothesis. The gB(100C) vesicles extended this observation. Again, we found two types of vesicles containing either postfusion gB or the compact gB separated from the membrane (Fig. 3F to I), with the compact gB-containing vesicles being approximately twice as abundant as the postfusion gB-containing vesicles. The FP-associated densities were apparent when the two conformations of gB were imaged by subtomogram averaging, at the same positions as shown for gB(81Y) (Fig. 3L and M). Compact gB(100C) was slightly shorter than its gB(81Y) counterpart (approximately 8 nm tall and 8 nm wide), but its separation from the membrane was the same (approximately 4 nm). The altered compact conformations (separated from the membrane) may represent an intermediate conformation of gB in its path to postfusion, as these constructs have fusogenic phenotypes that are similar, albeit not identical, to those of WT gB (34).

In conclusion, by using gB(81Y) and gB(100C) constructs, we have confirmed that the compact structures on the surface of vesicles are indeed gB. The position of the extra FP densities on gB allowed us to locate the end of domain IV and the beginning of the N terminus (domain VI) at the center and toward the outside of intermediate gB, as suggested by Gallagher et al., and places domain VI at the top of postfusion gB.

### Localization of domain II-expressing gB(470Y) and gB(81C-470Y).

**FIG 4:**
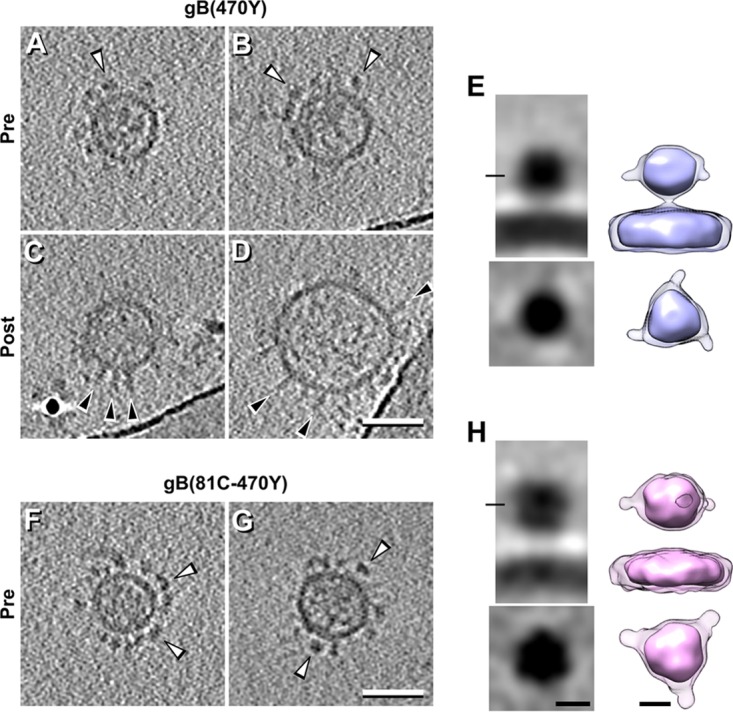
Microvesicles expressing full-length gB(470Y) or gB(81C-470Y) imaged by cryo-ET and subtomogram averaging. (A to E) Central tomographic sections (A to D) and subtomogram averaging (E) of microvesicles expressing full-length gB(470Y). (F to H) Central tomographic sections (F and G) and subtomogram averaging (H) of microvesicles expressing full-length gB(81C-470Y). The distribution of the subtomogram averaging panels is the same as that shown for WT gB in Fig. 2. To visualize the FP densities of these constructs, a lower threshold was used (lighter color in both averages). Bars: A to D, F, and H, 50 nm; E and H, 5 nm. White arrowheads indicate compact gB; black arrowheads indicate post-fusion gB.

Using the same approach, we took advantage of a form of gB with a YFP insertion at 470, gB(470Y), to elucidate the location of domain II in prefusion/intermediate gB (Fig. 4). Of note, residue 470 was not resolved in the neutral pH gB crystal structures (11, 12), suggesting that it is located in a flexible loop of the central lobe of postfusion gB. According to the prefusion gB model of Gallagher et al. (34), amino acid 470 should be located at the top of the prefusion form of gB, close to the 3-fold symmetry axis (Fig. 1C). However, the model of Zeev-Ben-Mordehai et al. (30) locates this residue close to the viral membrane at a position close to domain IV but offset by 60° (Fig. 1B).

Since gB(470Y) exhibits reduced cell surface expression (34), it was not surprising that it produced fewer vesicles. As with gB(81Y) and gB(100C), many of these vesicles contained only a compact conformation of gB. In fact, vesicles displaying the compact conformation were three times as abundant as those containing the postfusion form. Subtomogram averaging of the compact conformation of gB(470Y) showed that its size was similar (approximately 8 nm tall and 8 nm wide) to that of compact gB(100C) but was closer to the membrane (approximately 2 nm versus approximately 4 nm). Additionally, the FP density was present at the middle of gB, pointing toward the side, locating domain II in that general area (Fig. 4E). Because of the small number of postfusion gB(470Y) vesicles, we could not calculate a subtomogram average for this conformation.

We also imaged a gB construct containing two FPs (81C-470Y) (Fig. 4F to H). This construct was of special interest since residues 81 and 470 are not close to each other in the postfusion structure (residue 81 in domain VI is located at the top of postfusion gB, while residue 470 in domain II is at the center of postfusion gB), but fluorescence resonance energy transfer (FRET) experiments showed that the two fluorophores were within FRET distance (<5 nm) in the prefusion conformation (34). In fact, according to the prefusion models of Zeev-Ben-Mordehai et al. and Gallagher et al. and the subtomogram averages of gB(81Y) and gB(470Y), we expected the amino acid 81 and 470 FPs in gB(81C-470Y) to be too close to be separated in our 3D maps. Subtomogram averaging of gB(81C-470Y) in the putative prefusion conformation showed a globular domain approximately 9 nm tall and 9 nm wide, separated approximately 4 nm from the membrane (Fig. 4H). However, we could not distinguish independent densities for the 81 and 470 FPs in gB(81C-470Y). Similar to experiments with gB(470Y), few dually labeled postfusion gB molecules were found. Therefore, subtomogram averages could not be calculated for this conformation.

These experiments locate domain II toward the side of prefusion gB, in a location conflicting with respect to its proposed locations in both prefusion models (Fig. 1B and C). Possible reasons for this are described in the Discussion.

### Localization of domain I-coexpressing full-length gB with the SS55 Fab.

To localize the position of domain I in the compact gB structure, we took advantage of the SS55 Fab, which is a potent, conformation-specific neutralizing antibody with a known epitope. Its binding site was determined to be within domain I via negative-staining EM experiments with postfusion gB incubated with the SS55 Fab (23). In addition, MAb-resistant (*mar*) viruses for SS55 contain mutations at residues 199, 203, and 335 within domain I (23). To ensure that we labeled as many compact gB molecules as possible, we coexpressed full-length gB and recombinant SS55 Fab rather than adding purified Fab to the vesicle preparations. We therefore reasoned that coexpression of SS55 with gB would possibly trap gB in a prefusion conformation and prevent its conversion to the postfusion form. A similar approach was previously applied to lock a truncated soluble prefusion form of the fusion protein of respiratory syncytial virus (40). According to the prefusion gB model of Gallagher et al., the SS55 epitope should be located in the middle of the lower half of prefusion gB with this domain facing outward (close to residue 103 but offset by 60°; Fig. 1C). However, the model of Zeev-Ben-Mordehai et al. presumed that the SS55 epitope would be at the top of prefusion gB, buried within the contact point between protomers at the 3-fold symmetry axis (Fig. 1B).

**FIG 5:**
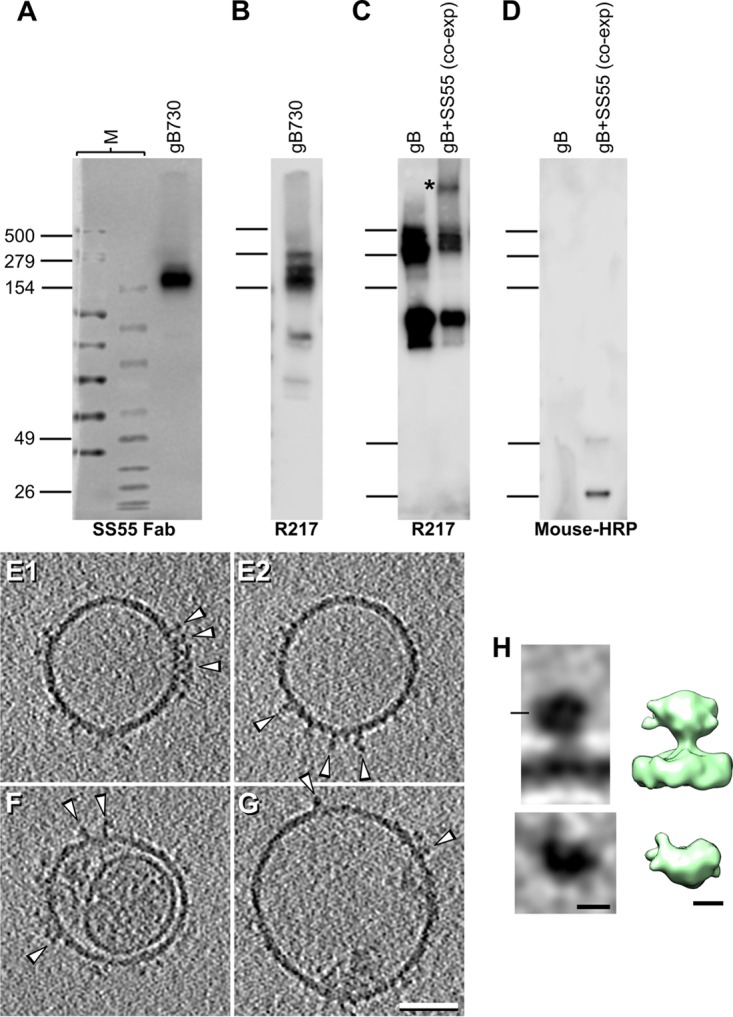
Microvesicles expressing full-length gB coexpressed with SS55 Fab. (A to D) Characterization of SS55 Fab produced in mammalian cells. (A and B) Purified truncated gB730 (A, rightmost lane) was run on a 4 to 12% gradient gel along with high-molecular-weight (A, left lane M) and broad-range (A, right lane M) molecular size markers in duplicate. One membrane was probed with supernatant from cells transfected with plasmids encoding the SS55 heavy and light chains (A). The values to the left are molecular sizes in kilodaltons. The second membrane (B) was probed with PAb R217. (C) A small proportion of gB present on microvesicles forms a higher-molecular-weight complex when coexpressed with SS55 Fab plasmids (asterisk) that is absent when Fab plasmids are not included in the transfection (lane 1). SS55 Fab can be detected under denaturing conditions in the gB microvesicle preparations (D). HRP, horseradish peroxidase. (E to H) Central tomographic sections (E to G) and subtomogram averaging (H) of microvesicles expressing full-length gB coexpressed with SS55. The distribution of the subtomogram averaging panels is the same as that shown for WT gB in Fig. 2. Bars: E to G, 50 nm; H, 5 nm. Arrowheads indicate compact gB.

We first sequenced the SS55 hybridoma and cloned the heavy and light chains into the pcDNA3.1 expression vector as described in Materials and Methods. As evidenced by Western blotting, the medium from 293T cells that had been cotransfected with these plasmids recognized purified soluble gB730 (Fig. 5A) similarly to gB recognized by a gB PAb (Fig. 5B). This confirmed that the SS55 Fab was indeed produced in mammalian cells and that it recognized gB. We then isolated vesicles from cells transfected with full-length gB alone or coexpressed with the plasmids encoding SS55 Fab. As shown in Fig. 5C, the presence of the SS55 Fab resulted in an increase in the size of a portion of gB (asterisk), indicative of a gB-SS55 Fab complex. The presence of the Fab in this sample was further confirmed by Western blotting (Fig. 5D).

The gB-SS55 Fab microvesicles contained almost exclusively the compact form of gB when imaged by cryo-ET (Fig. 5E to G). In fact, we found 10 times as many vesicles displaying compact forms as vesicles displaying postfusion gB. The few vesicles containing postfusion gB appeared not to be bound to SS55. This is consistent with our hypothesis that binding of SS55 prevents the prefusion-to-postfusion transition of gB. Subtomogram averaging of the compact form of gB showed a globular domain approximately 8 nm tall and 8 nm wide containing an extra density, corresponding to the Fab, at the side of gB (Fig. 5H). Of note, each gB molecule contained only one additional density, preventing the application of 3-fold symmetry. Compact gB is separated only approximately 2 nm from the membrane, and therefore it appears that SS55 traps gB in a conformation that differs from that of prefusion WT and the gB(81Y) and gB(100C) intermediates, but in a form similar to that of gB(470Y). We conclude that domain I is located on the side of an intermediate form of gB, as proposed in the model of Gallagher et al. To our knowledge, this is the first time a transfected Fab has been used to trap a membrane-bound protein in a conformation other than a first prefusion form and highlights the potential for using neutralizing antibodies to trap gB in intermediate conformations.

### Localization of the fusion loops by incubation of full-length gB with a PAb to fusion loop 2.

One of the features common to all enveloped virus fusion proteins is that their fusion loops or fusion peptides are masked or point toward the viral membrane in their prefusion conformation (2, 41). Therefore, the most striking feature of the gB prefusion model by Zeev-Ben-Mordehai et al. is that the fusion loops point away from the viral membrane. In contrast, the fusion loops point toward the viral membrane in the model of Gallagher et al.

We previously prepared a PAb, R240, directed against fusion loop 2 of gB by hyperimmunizing rabbits with a peptide spanning this loop (residues 252 to 271) (23). To reduce the number of nonspecific antibodies present in the polyclonal serum, we purified R240 by passing it though a column containing immobilized gB730 (see Materials and Methods).

**FIG 6:**
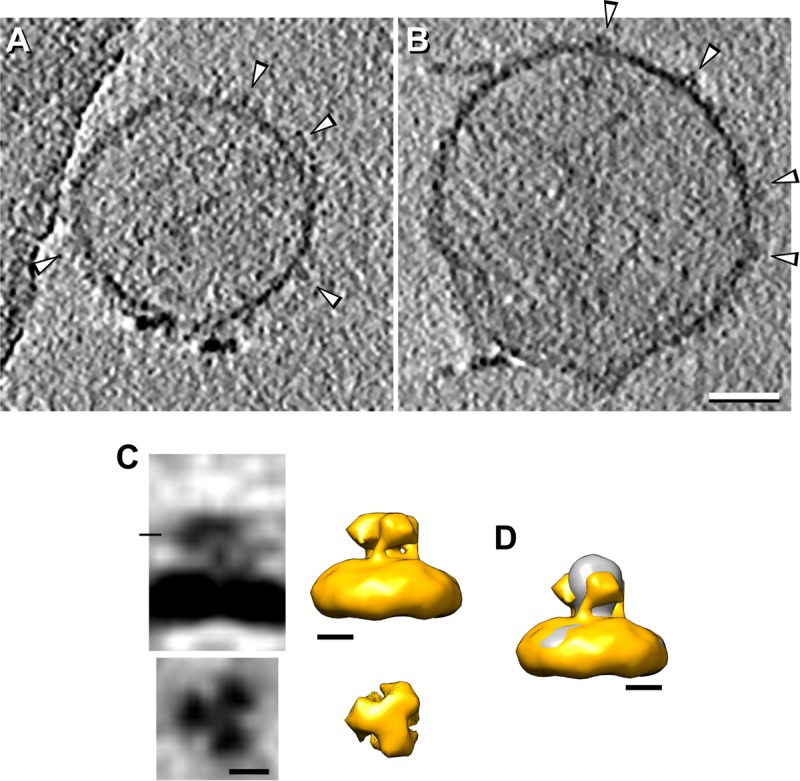
Microvesicles expressing full-length WT gB incubated with an antibody against fusion loop 2. (A to C) Central tomographic sections (A and B) and subtomogram averaging (C) of microvesicles expressing full-length gB incubated with antibody R240 against fusion loop 2. The distribution of the subtomogram averaging panels is the same as that shown for WT gB in Fig. 2. (D) Isosurface rendering of WT gB (gray) together with the WT gB incubated with R240 (gold). Bars: A and B, 50 nm; C and D, 5 nm. Arrowheads indicate compact gB.

To locate the fusion loops in full-length gB, we incubated microvesicles containing gB with a 10 M excess of purified R240. As shown in Fig. 6, subtomogram averaging of this sample shows extra densities at the bottom/side of gB, locating the fusion loops close to the viral membrane. Of note, fusion loop 2 is located at the bottom of domain I in postfusion gB, extending up to half of its length. Therefore, the localization of the extra densities in this experiment is in agreement with the model of Gallagher et al.

## DISCUSSION

In this study, we characterized full-length gB expressed on the surface of HIV pseudotyped particles and microvesicles secreted from 293T cells. We found that WT gB was present in both pre- and postfusion conformations on the membrane surfaces. Using preparations of microvesicles, we also expressed gB molecules with FP insertions in different domains of the glycoprotein. Additionally, we coexpressed gB with the neutralizing antibody to SS55. Finally, we incubated gB with a purified PAb directed against the gB fusion loops. These approaches produced different structures of gB that we interpret to be intermediates in gB’s transition from the prefusion to the postfusion conformation. To use FPs, Fabs and antibodies as landmarks, we used cryo-ET and subtomogram averaging. This approach allowed us to locate different domains of gB in these intermediate conformations and extrapolate their location in prefusion gB. We have further characterized the antigenic properties of these constructs, confirming that the epitopes for a subset of our antibody collection are preserved in the gB constructs studied.

**FIG 7:**
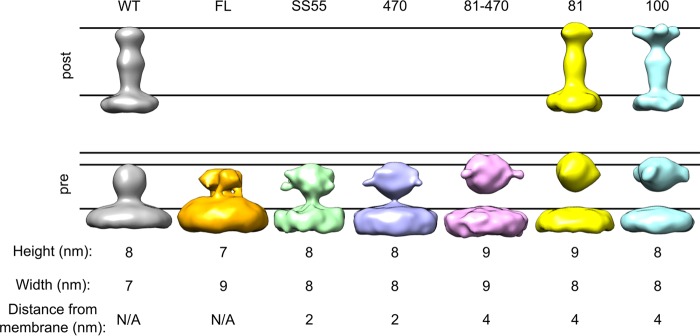
Summary of subtomogram averages. Subtomogram averages calculated in this study are shown together for comparison. The lines in the postfusion conformation show the top of the membrane and the top of postfusion WT gB. The lines in the prefusion conformation show the top of the membrane, the top of prefusion WT gB, and the top of gB(81C-470Y). Measurements and distances to the membrane of the gB prefusion conformations are included. FL, fusion loop; N/A, not applicable.

A summary of all of the averages produced in this study is shown in Fig. 7. We conclude the following.

### The compact form is prefusion gB.

In both microvesicles and pseudotyped HIV particles, we could find only two conformations of gB, a compact form and the postfusion form. Using three independent approaches, we labeled the compact form, which allowed us to identify it as gB. It has been previously shown that cells expressing HSV-1 gB can fuse with cells expressing the gD receptor and gH/gL in the presence of soluble gD (42). Therefore, we take this result to indicate that a prefusion form of HSV-1 gB is stable in the absence of gH/gL and gD, even though it has been shown that gB and gH/gL from human cytomegalovirus might form a stable complex in virions (43) and that the cytoplasmic tail of HSV-1 gH/gL has been proposed to be involved in gB activation (44). Since, in the absence of a target membrane, gB can adopt the postfusion conformation without gH/gL and the interaction between gH/gL and gB appears to occur after gB interacts with the target membrane (24), we speculate that the function of gH/gL is to provide the energy required for membrane merging during the fusion process.

### Forms of gB containing FP insertions represent fusion intermediates.

We reasoned that gB containing FP insertions would be an optimal way to locate the different domains in prefusion gB. This was based on two facts, (i) that FP molecules are bulky enough to be seen in subtomogram averages, even at low resolution, and (ii) that the genetic encoding of the FPs guarantees that all gB molecules contain three copies of the tag. However, we previously showed that the gB molecules containing FP insertions behave differently from WT gB in terms of fusion when expressed on the surface of eukaryotic cells (34). Therefore, we suggest that they could present a somewhat different conformation (not surprising, since each FP is approximately 27 kDa). The results obtained with the FP insertions support our hypothesis, since WT gB was in close contact with the microvesicle membrane, while all gB-FP samples, especially N-terminal FP insertions [gB(81Y), gB(100C), and gB(81C-470Y)], showed a clear separation (i.e., the stalk connecting the globular domain of gB to the membrane was not visible in the averages). For other enveloped viruses, e.g., influenza virus, it has been suggested that the fusion protein adopts an intermediate conformation in which the molecule is completely extended (termed the extended intermediate), so that the transmembrane region and the fusion peptides/loops are on opposite sides of the molecule (41, 45). We propose that the gB-FP compact averages represent partially extended intermediates that correspond to conformational changes in domain III or V, which likely experience extensive conformational changes during the fusion process, as predicted for the equivalent domains in VSV-G. We acknowledge, however, that the averages could also represent altered prefusion conformations caused by the FP insertions.

### Localization of the N terminus in prefusion gB.

As discussed above, the structure of the N terminus is unknown and it has been suggested that it is flexible (12). In addition to being the site of binding of several neutralizing MAbs (27), it contains the heparan sulfate binding region, which is required for efficient virus entry (46). This suggests that the N-terminal region is accessible on prefusion gB (i.e., it is on the outside of prefusion gB). This is in agreement with our averages of gB(81Y) and gB(100C) that locate the beginning of the N terminus (domain VI) at the center and toward the outside of intermediate gB. Additionally, our data clearly show that FP insertions in the N terminus [gB(81Y), gB(100C), and gB(81C-470Y)] increase the separation of the globular prefusion domain of gB from the membrane. This suggests that the N terminus is involved in interactions of prefusion gB with the viral membrane, locating the N terminus at the base of the prefusion conformation. A possible explanation for these observations is that the N terminus stretches from the side of prefusion gB to the bottom of prefusion gB, as has been previously suggested for postfusion gB (23). While this explanation is speculative, since, as mentioned above, the insertion of FPs alters the conformation of gB, this model agrees with the fact that the antibody H1817, whose epitope is located in the N terminus (residues 31 to 43) (27) competes with DL16, whose epitope is located in domain V (23).

### gB domains remain constant during gB rearrangement.

Compared to gB, much more is known about VSV-G, another class III fusion protein. Structures of G include prefusion (32), postfusion (31), and monomeric intermediate conformations (47–49). These structures revealed that most domains of G suffer only minor conformational rearrangements during the transition between pre- and postfusion. On the basis of these observations, it has been hypothesized that the prefusion domains of gB are similar to its postfusion domains. In fact, the prefusion gB models of both Zeev-Ben-Mordehai et al. and Gallagher et al. (30, 34) were generated by using this hypothesis, although no experimental data have confirmed it. To test this hypothesis, we employed a nondenaturing electrophoresis-Western blot assay procedure (39), probing strips of the blot with MAbs targeting domains I (SS55), II (C226), and IV (SS10) (Fig. 3A). As all forms of gB were recognized similarly by the three MAbs, this result shows that their corresponding epitopes remain constant in all constructs. Therefore, we suggest that the overall conformation of each domain remains stable, as has been previously suggested (30, 34). However, we acknowledge the fact that our preparations contain gB in both prefusion and postfusion conformations. Further analysis of a sample containing exclusively prefusion gB is required to confirm if gB domains are indeed similar in its prefusion and postfusion conformations.

### Fit of gB averages with current gB prefusion models.

**FIG 8:**
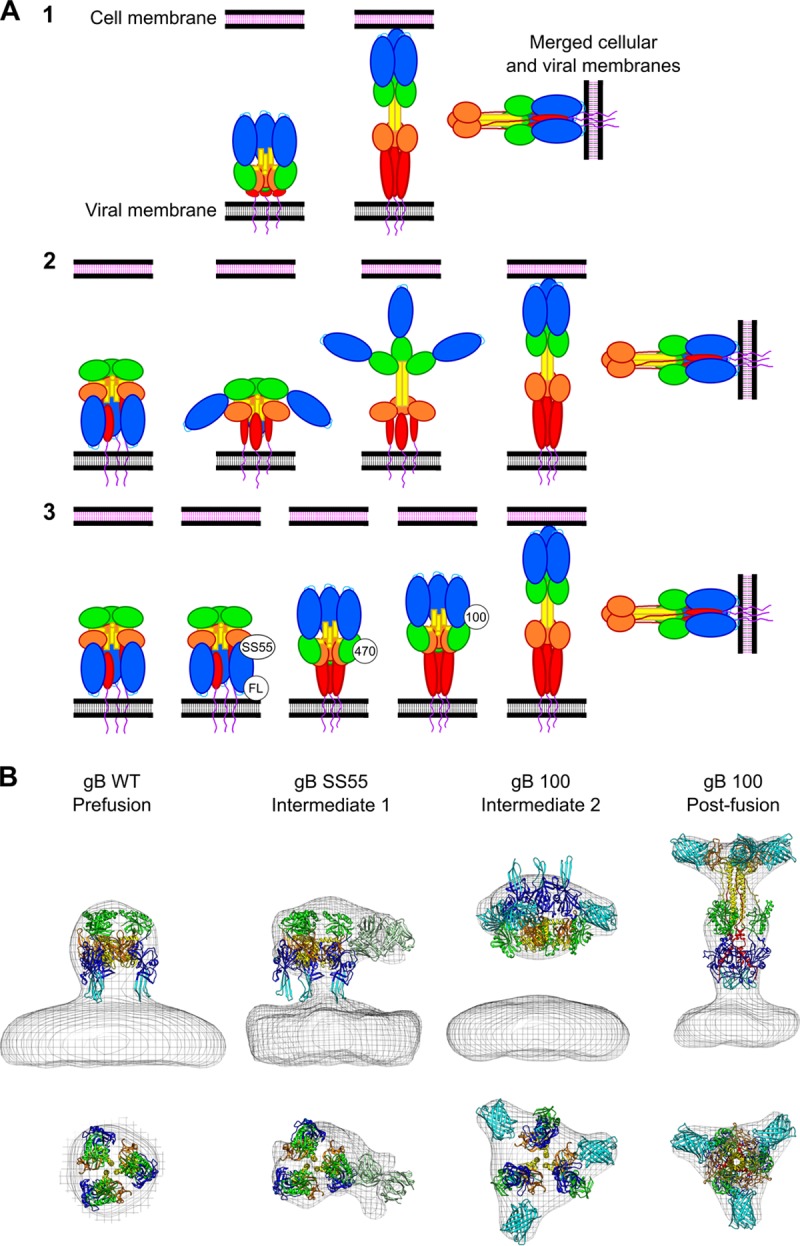
Model of fusion. (A) Transitions of gB from the prefusion to the postfusion conformation. According to hypothesis 1, fusion loops point up in the prefusion conformation. According to hypothesis 2, fusion loops point down in the prefusion conformation and the gB compact structure does not contain fusion loops pointing down. According to hypothesis 3, the model starts with the fusion loops pointing toward the viral membrane with an intermediate-containing compact form with the fusion loops pointing up. (B) Models of prefusion, intermediate, and postfusion gB fitted into representative gB subtomogram averages from this study and according to hypothesis 3. The prefusion model of Gallagher et al. is fitted into prefusion WT gB and in gB coexpressed with the SS55 Fab. A chimeric model using domains I and II from the model of Zeev-Ben-Mordehai et al. and domains III and IV from the model of Gallagher et al. is fitted into 100Y intermediate average. Postfusion gB from Stampfer et al. (12) (PDB code 3nw8) is fitted into postfusion gB(100C). GFP molecules and a Fab are included in the gB SS55 and gB(100C) panels, respectively.

On the basis of the two current prefusion gB models, we envision three hypotheses to explain the prefusion-to-postfusion transition of gB (Fig. 8A). In hypothesis 1, according to the model proposed by Zeev-Ben-Mordehai et al. (30), the fusion loops point away from the viral membrane in the initial prefusion conformation. Therefore, to reach an extended intermediate conformation, gB would first undergo an extension to reach the target membrane. This extension could be mediated by a refolding of domain V. It has been shown that the MPR (amino acids 730 to 773, for which there is no available structure) regulates the exposure of the fusion loops (50). Thus, the MPR could also be involved in this extension. Following the extended intermediate, the molecule would need to fold back upon itself (experiencing an “inversion”), placing the fusion loops at the same end as the transmembrane domains and leading to fusion (Fig. 8A1). In hypotheses 2, if the model proposed by Gallagher et al. is correct, the fusion loops would point toward the viral membrane in the prefusion conformation. Thus, gB could reach the extended intermediate by experiencing an initial inversion at the same time as the fusion loops are relocated to the top of the molecule. In this sequence of events, the compact structure would never contain fusion loops that point away from the viral membrane. This inversion would be mediated by the formation of the central stalk seen in the postfusion structure. From this extended intermediate, a second inversion (like the one described above) would induce fusion (Fig. 8A2). Alternatively, in hypothesis 3, according to the model of Gallagher et al., prefusion gB could experience an initial inversion, with domain II pivoting close to the viral membrane and acquiring a conformation similar to the one proposed by Zeev-Ben-Mordehai et al. (i.e., with the fusion loops pointing away from the viral membrane). Extension of the structure and formation of the central helical stalk would follow as a distinct kinetic step, which could resolve to membrane fusion through the second inversion described above (Fig. 8A3).

While we acknowledge that higher-resolution averages are needed to unequivocally unravel gB’s mechanism of fusion, our results, do not support hypothesis 1. Consistent with hypotheses 2 and 3, extra densities from the fusion loop antibodies are found at the side of gB, close to the viral membrane (Fig. 6) and not at the top of the molecule, as proposed in hypothesis 1. Additionally, the SS55 density is located at the middle lower half of prefusion domain facing outward (Fig. 5), again consistent with hypotheses 2 and 3, while hypothesis 1 predicts that this epitope is located at the top of the molecule, close to the 3-fold symmetry axis.

To discern between hypotheses 2 and 3, we then considered the gB FP averages. Specifically, our data could not reconcile the gB(470Y) average, seen toward the center of gB and facing outward (Fig. 4), with hypothesis 2, which locates residue 470 at the top of prefusion gB. We therefore propose that prefusion gB, with the fusion loops at the bottom, undergoes an initial inversion while retaining an overall compact conformation, following the pathway described for hypothesis 3. This initial inversion could be mediated by a conformational change in domain V (or the MPR; see above), adopting a compact conformation with the fusion loops pointing away from the viral membrane, reconciling our data with data described by Zeev-Ben-Mordehai et al. Following this inversion, domain III would extend, allowing gB to adopt the extended intermediate conformation, and a second conformational change in domain V will finally convert gB into its postfusion conformation, allowing membrane fusion (Fig. 8B).

In summary, we have used vesicles to express WT gB and a series of FP-modified forms of gB, as well as anti-gB antibodies, to study different forms of gB by cryo-ET. We have located the positions of specific residues and domains on postfusion gB and what we believe to be the prefusion and possible intermediate forms. Our data show for the first time how gB might transition from its starting place to its final stable postfusion form.

## MATERIALS AND METHODS

### Cells and plasmids.

293T cells (ATCC CRL-3216) were grown in Dulbecco’s modified Eagle’s medium (DMEM; Gibco) supplemented with 10% fetal bovine serum (FBS) and 100 µg/ml penicillin-streptomycin at 37^o^C in 5% CO_2_.

gB_1_ (pPEP98) was a gift from P. Spear (51). Fluorescent constructs gB(81Y) (pJG1040), gB(100C) (pJG1049), gB(470Y) (pJG1025), and gB(81C-470Y) (pJG1026) were described previously (34). For pseudotyped HIV particle production, psPAX2 was obtained through the NIH AIDS Reagent Program, Division of AIDS, NIAID, NIH (catalog no. 11348) from D. Trono (36).

### SS55 Fab sequencing and expression.

Total RNA was extracted from frozen SS55 hybridoma. cDNA was synthesized, and PCR was performed to amplify the variable and constant regions of the antibody. The fragments were cloned into standard cloning vectors separately and sequenced (GenScript, Piscataway, NJ). The light and heavy chains were reconstructed by overlap extension PCR. For light chain reconstruction, the primers used were TTGGTACCATGAGTGTGCCCACTC (primer A), TGTTCAAGAAGCACACGACTG (primer B), GCTTCTTGAACAACTTCTACCCCAAAGAC (primer C), and TAATCTCGAGCTAACACTCATTCCTG (primer D). For heavy chain reconstruction, the primers used were TTGGTACCATGAACTTCGGGCTC (primer A), CACTGTCACTGGCTCAGGG (primer B), GCCAGTGACAGTGACCTGGAACTC (primer C), and TAATCTCGAGTCAAATTTTCTTGTCCACC (primer D). The final PCR products were cloned into the pcDNA3.1 expression vector, resulting in pRC1058 (SS55 heavy chain) and pRC1059 (SS55 light chain). The correct orientation of constructs was confirmed by sequencing.

### Transfection. (i) Pseudotyped HIV particles.

293T cells (2 × 10^6^/well) were seeded onto six-well plates. One microgram of psPAX2 and 2 µg of pCAGGS, WT gB_1_, or FP-tagged gB were transfected with 10 µl of Lipofectamine 2000 (Invitrogen). All transfections were performed at 37°C, with the exception of gB(470Y) and gB(81C-470Y). For these constructs, cells were transfected for 5 h at 37°C and then transferred to 32°C (34).

Before transfection, growth medium from each well was replaced with 2 ml of fresh DMEM containing 10% exosome-depleted FBS with no antibiotics. To obtain exosome-depleted FBS, FBS was centrifuged at 28,000 rpm at 4 C for at least 16 h with an SW-41 Ti rotor. Supernatants were collected and filtered (0.22-µm pore size). The pelleted exosomes were discarded.

### (ii) Microvesicles.

The transfection conditions used were similar to those used for pseudotyped HIV particle production, but the psPAX2 plasmid was excluded from the mixture.

### (iii) gB-SS55 Fab coexpression.

One microgram each of gB_1_, SS55 heavy chain (Hc), and SS55 light chain (Lc) plasmids was transfected into 293T cells.

### Purification of full-length-gB-expressing particles.

Cell media were collected from transfected cells at 48 and 96 h posttransfection and clarified by low-speed centrifugation (1,000 rpm, 4ºC, 10 min). Supernatants devoid of cell debris were filtered through a 0.45-µm-pore-size filter. Vesicles were purified by ultracentrifugation through a 5-ml 20% sucrose-HBS cushion (20% sucrose in distilled H_2_O with 20 mM HEPES, 150 mM NaCl, filter sterilized) at 28,000 rpm at 4ºC for 2 h with an SW-41 Ti rotor. HIV pseudoparticles/microvesicle pellets were resuspended overnight in HBS at 4°C with gentle shaking.

### Purification of fusion loop PAb.

The procedure used to make a soluble gB-CNBr Sepharose 4B column was described previously (23).

The gB_2_(727t) column was washed extensively with TS buffer (10 mM Tris-HCl [pH 7.2], 0.5 M NaCl) before being loaded with 10 mg of total anti-fusion-loop IgGs. The flowthrough was collected and reloaded onto the column five times; this was followed by washing with TS buffer. The gB-specific IgGs were eluted with 3 M KSCN. The eluted sample was dialyzed against phosphate-buffered saline and concentrated with centrifugal filter units (Millipore). For cryo-ET, the purified PAb was mixed with WT gB microvesicles at a 10 M excess overnight at 4°C.

### Western blotting.

A 2.5-µl portion of each microvesicle sample was run on 10% or 4 to 12% gradient Tris-glycine gels (Novex) under native or denaturing conditions (as indicated) and subjected to Western blotting. To probe for gB, PAb R217 [generated against truncated gB_1_(730) as described previously (52)] or MAbs (27) were used, as indicated. The fluorescent tags were detected with anti-GFP polyclonal antibody ab6556, and HIV Gag protein was detected with p24 MAb ab9071, both from Abcam, Inc. All secondary antibodies (goat anti-mouse or goat anti-rabbit) were coupled to horseradish peroxidase (Cell Signaling).

### Cryo-ET.

Grids containing microvesicles or HIV pseudotyped particles were prepared as previously described (53). In brief, samples were mixed 2:1 with a suspension of bovine serum albumin (BSA)-coated 10-nm colloidal gold particles (Aurion, Wageningen, The Netherlands) that served as fiducial markers, and 4-µl drops were then applied to R2/2 holey carbon grids (Quantifoil; SPI, West Chester, PA). Samples were blotted and vitrified by plunge-freezing in liquid ethane with a Vitrobot (FEI, Hillsboro, OR) or a Leica EM GP Automatic Plunge Freezer (Leica Microsystems, Inc., United Kingdom). Grids were then transferred under cryogenic conditions to a specimen holder (type 626; Gatan, Warrendale, PA) for data acquisition. A Tecnai-12 transmission electron microscope (FEI) operated at 120 kV was used to record single-axis tilt series. Imaging was done with an energy filter (GIF 2002; Gatan) operated in zero-loss mode with an energy slit width of 20 eV. Tilt series were acquired by serial EM (54). Images were recorded on a 2,048- by 2,048-pixel charge-coupled device camera (Gatan) in 2° increments over an angular range of approximately −60° to +60° at a nominal magnification of ×38,500 (0.78-nm pixel size) or ×53,600 (0.56 nm/pixel). The electron dose per image was approximately 1 e^−^/Å^2^, adding to a cumulative dose of approximately 70 e^−^/Å^2^ per tilt series. The nominal defocus was −4 μm, corresponding to a first contrast transfer function zero at 3.7 nm^−1^.

### Tomogram reconstruction and subtomogram averaging.

Tilt series were reconstructed with the Bsoft package (55). Individual gB molecules were located on microvesicles or pseudotyped particles denoised with an anisotropic nonlinear diffusion filter (56) and were extracted from the corresponding raw subtomograms. The center of each microvesicle/pseudotyped particle was used to calculate the initial orientation of each gB molecule. Alignment procedures were performed with routines from Bsoft, modified as needed and wrapped into Python scripts. An average of all of the selected subtomograms, obtained by using the initial orientations described above, was used as the initial model for each gB sample studied. Threefold symmetry was used for all averages (WT gB, gB-FPs, and gB incubated with the fusion loop antibodies), except for the gB sample coexpressed with the SS55 neutralizing antibody. Final classification and averaging were performed by the maximum-likelihood method (57). The average resolution was 5 nm, as calculated in terms of Fourier shell correlation (0.3 threshold). Table S1 summarizes the number of particles and resolutions used for each average. Fitting of the atomic models into the subtomogram averages was done manually with the UCSF Chimera package (58).

10.1128/mBio.01268-17.1 TABLE S1  Summary of the number of particles used to calculate the subtomogram averages for the different samples analyzed in this study and the resolutions achieved. Download TABLE S1, DOCX file, 0.02 MB. Copyright © 2017 Fontana et al. 2017 Fontana et al. This content is distributed under the terms of the Creative Commons Attribution 4.0 International license.
