# Supplementary material for: The Fusion Loops of the Initial Prefusion Conformation of Herpes Simplex Virus 1 Fusion Protein Point Toward the Membrane
Source: mBio. 2017 Aug 22;8(4):e01268-17. doi: 10.1128/mBio.01268-17 (PMC5565971; doi:10.1128/mBio.01268-17)
Supplement: TABLE S1 [file mbo004173444st1.docx]

| **Sample** | **Conformation** | **# of selected subtomograms** | **# of subtomograms used for final reconstruction** | **Resolution (nm)** |
| --- | --- | --- | --- | --- |
| **gB WT microvesicles** | Pre-fusion | 197 | 197 | 4.8 |
|  | Post-fusion | 225 | 184 | 4.6 |
| **gB WT pseudo-typed HIV** | Pre-fusion | 46 | 46 | 6.0 |
|  | Post-fusion | 33 | 33 | 5.0 |
| **gB(81Y)** | Pre-fusion | 155 | 121 | 5.9 |
|  | Post-fusion | 106 | 82 | 5.6 |
| **gB(100C)** | Pre-fusion | 206 | 152 | 4.5 |
|  | Post-fusion | 227 | 173 | 4.4 |
| **gB(470Y)** | Pre-fusion | 179 | 179 | 4.1 |
| **gB(81C-470Y)** | Pre-fusion | 66 | 66 | 5.4 |
| **gB-SS55** | Pre-fusion | 78 | 58 | 4.2 |
| **gB-FL** | Pre-fusion | 65 | 65 | 9.5 |

**Supp. Table 1.** Summary of the number of particles used to calculate the subtomogram averages for the different samples analyzed in this study, and the resolutions achieved.
